# Sometimes Resigned, Sometimes Conflicted, and Mostly Risk Averse: Primary Care Doctors in India as Street Level Bureaucrats

**DOI:** 10.34172/ijhpm.2020.206

**Published:** 2020-10-28

**Authors:** Sudha Ramani, Lucy Gilson, Muthusamy Sivakami, Nilesh Gawde

**Affiliations:** ^1^School of Health Systems Studies, Tata Institute of Social Sciences, Mumbai, India.; ^2^Division of Health Policy and Systems, University of Cape Town, Cape Town, South Africa.; ^3^Department of Global Health and Development, London School of Hygiene and Tropical Medicine, London, UK.; ^4^Center for Health and Social Sciences, School of Health Systems Studies, Tata Institute of Social Sciences, Mumbai, India.

**Keywords:** Street Level Bureaucracy, Policy Implementation, Primary Health, India, Doctors

## Abstract

**Background:** In this study, we use the case of medical doctors in the public health system in rural India to illustrate the nuances of how and why gaps in policy implementation occur at the frontline. Drawing on Lipsky’s Street Level Bureaucracy (SLB) theory, we consider doctors not as mechanical implementors of policies, but as having agency to implement modified policies that are better suited to their contexts.

**Methods:** We collected data from primary care doctors who worked in the public health system in rural Maharashtra, India between April and September 2018 (including 21 facility visits, 29 in depth interviews and several informal discussions). We first sorted the data inductively into themes. Then we used the SLB theoretical framework to categorise and visualise relationships between the extracted themes and deepen the analysis.

**Results:** Doctors reported facing several constraints in the implementation of primary care- including the lack of resources, the top-down imposition of programs that were not meaningful to them, limited support from the organization to improve processes as well as professional disinterest in their assigned roles. In response to these constraints, many doctors ‘routinized’ care, and became resigned and risk-averse. Most doctors felt a deep loss of professional identity, and accepted this loss as an inevitable part of a public sector job. Such attitudes and behaviours were not conducive to the delivery of good primary care.

**Conclusion:** This study adds to empirical literature on doctors as Street Level Bureaucrats in lower and middle income countries. Doctors from these settings have often been blamed for not living up to their professional standards and implementing policies with rigour. This study highlights that doctors’ behaviours in these settings are ways through which they ‘cope’ with their loss of professional identity and organizational constraints; and highlights the need for appropriate interventions to counter their weak motivation.

## Background

Not all policy rhetoric translates into practice as originally intended. Even when top-down policy mandates are firmly imposed, gaps in implementation of policies can occur in the frontlines of service delivery. Why such gaps occur – and what can be done about these – have been questions of interest to policy analysts. Theories such as Lipsky’s Street Level Bureaucracy (SLB)enable policy analysts to deepen their understanding of such gaps better.^1^ SLB theory posits that people at the frontline do not mechanistically comply to all imposed policy mandates.^1-3^ Nor do they implement these mandates in a vacuum. Instead, they implement a version of the policy mandate that appears to fit in with their everyday realities and contextual constraints.^1-2,4^ To cope with the wide range of contextual complexities they encounter, people at the frontline of policy delivery develop informal routines and practices.^1-3,5,6^ These routines and practices can be called ‘*coping behaviours*’ and lead to policies being implemented in ways that are different from the written policy rhetoric. Hence, people in the frontline of policy delivery have been called ‘*Street Level Bureaucrats*’ who create ‘*policies as performed*.’^1^ It is this version of policies that the public ultimately experiences.

In this paper, we have drawn on SLB theory to examine how medical doctors in public primary health centers (a type of peripheral health facility) in one rural area in India delivered routine primary care. We have asked two specific questions:

1. What were the ‘*coping behaviours’* exhibited by doctors in primary health facilities in rural India?
2. In what ways did the professional identities of doctors vis-à-vis the organisational constraints they faced shape these ‘*coping behaviours’*?

This paper is an attempt to add to theory-driven, actor-centric, empirical studies in the field of health policy implementation in low- and middle-income countries (LMICs). There has been a growing interest in understanding frontline health workers behaviours (nurses, doctors, outreach workers and managers) in such settings.^7-12^ However, within LMIC literature, medical doctors working at the frontlines of care delivery have been less frequently been examined using SLB theory (some recent exceptions being Karadaghi and Willott,^13^ Gaede,^14^ Kelly^15^). In this paper, we have explicitly focussed on understanding the perspectives of medical doctors. This focus is particularly interesting since doctors often experience tensions between their twin identities as ‘professionals’ and as ‘bureaucrats,’ and struggle to balance these different identities in their actions.^16^

Another contribution of this study is its focus on the implementation of day-to-day primary care. Most studies from LMICs that have engaged with SLB theory focus on how staff in the frontline deal with newly instituted health policies. Very few studies have examined the implementation of policies long in existence and looked at routine practices within health systems (termed “coping” in this study) as important influencers of policy success on the ground.^6,16-17^ In this study, wehave treated the delivery of primary care by doctors as a “routine” policy, and have focussed on the decisions made by doctors in their day-to-day work.

This paper builds on a previously published paper that delineated broad contextual influences on primary health centers in India.^18^ Located in peripheral areas, these centers are intended to deliver preventive as well as basic curative care in the public health system (see Box 1 for a detailed background). The quality of care delivered by such facilities, and indeed, similar facilities in other LMICs, has been lamented repeatedly.^19^ Literature indicates that frontline workers such as doctors and nurses, in these settings, often exhibit disrespectful attitudes towards patients and do not live up to professional expectations.^20-22^ In this paper, we have endeavoured to move beyond blaming frontline workers for such actions and attitudes. Instead, taking one small rural area in India as a case, we have examined doctor’s behaviours as contextually situated actions; and have attempted to understand the nature of their behaviours as well as why such behaviours often persist in public systems of resource-constrained settings.

**Box 1. Primary Health Centers in India and the Role of Allopathic Doctors**

Primary health centers are peripheral health facilities within India’s tiered, pyramidal public health system. They are intended to be frontline institutions of care, delivering integrated preventive and curative care close to homes of people – and linking to specialty care through referral. These centers are considered as the first access points to a medical doctor in the public health system. One primary health center is intended for every 30, 000 population (20000 in tribal and hilly areas).

The doctor at a primary health center (in this context, having an undergraduate degree in allopathic medicine) is considered as a social physician, responsible for the health of the entire service area under a primary health center. As the medical officer-in-charge, the doctor also serves as the administrative head of the health center, and manages the support staff (nurses, laboratory technicians, pharmacist and other outreach workers). Other roles include the provision of primary-level clinical care, oversight of outreach care, implementation of all programs and schemes, and overall management of staff.

Source: Bulletin of rural health statistics, 2017^23^ and Indian Public Health Standards, 2012.^24^

## Methods

### Study Setting

This study was conducted in rural Maharashtra, a state in western India. Maharashtra is considered as one of the five ‘*high production’* states for allopathic doctors in India, having 52 medical colleges of which 23 are public.^25,26^ A one-year rural service stint is compulsory for all undergraduate medical students from public colleges and this policy, to some extent, has enabled the filling of allopathic doctors’ posts at primary health centers in the state (unlike many other Indian states).^27^ Despite filled posts, primary health centers in Maharashtra often do not serve as first access care to communities. A 2014 national survey found that in Maharashtra, only 7.5% of people seeking ambulatory care utilized primary-level tiers in the public health system.^28^

This study was undertaken in one small rural area. The name of the area as well as the district have been masked to protect the participants. This area had access to approximately 80 primary-level facilities, one district-level (secondary care) hospital and one non-for-profit tertiary care hospital. This area was chosen for the study since it had a mix of facilities with ‘good’ and ‘poor’ infrastructure (measured as per government quality standards) and posts for allopathic doctors was filled in most of the facilities. Process indicators from the health centers in this rural area for services like antenatal care and immunization were slightly better than that of state averages; and the area was considered to be overall, a ‘medium’ performing region (according to the state and district level authorities).

### Data Collection

We visited 21 health facilities and interviewed 29 public sector doctors from the rural area we selected (see Table 1 for details on data collection). We used the principle of maximum diversity to choose doctors for our interviews; we tried to include doctors of different ages and experiences, from facilities with good and not-so good infrastructure and from remote as well easy-to-access facilities. We purposively sampled four female doctors who were available in this region (the lack of female doctors in peripheral areas is an important concern across rural areas in India^29^). We also sought the opinion of 5 doctors who had earlier worked in primary care roles and had now moved on to other roles- to offer comparative perspectives on the different roles they performed.

**Table 1:** Details of Data Collection

**Profile of Study Participants**
Total	N = 27^a^
Education	Allopathic undergraduate degree: 22, Post-graduate degree: 5
Gender	Male: 23, Female: 4
Age	Average age of participants: 39.2 Less than 25 years of age: 4 25-45 years of age:16 More than 45 years of age: 7
Years of experience	Average years of experience of participants: 12.1 Less than 5 years of experience: 6 5-15 years of experience:12 More than 15 years of experience: 9
Place of work	Higher tiers of the system: 5^b^ Primary health centers – easy to access (less than 10 km from state highway): 9 Primary health centers – remote (more than 10 km from state): 13
Key themes in the topic guide Doctor’s roles at the primary health centers Doctors professional goals and dreams Stories about their best experiences at the primary health center (while implementing programs or doing outpatient care) Stories about challenging experiences and circumstances Stories about professional colleagues in other places Examples of situations that doctors faced day-to-day and how they dealt with these Doctors’ position in the organizational hierarchy and relationships (stories or experiences pertaining to interactions with seniors) Doctors’ relationships with support staff (examples of interactions with support staff) Doctors’ relationships with patients and the community (examples of interactions with the community)	

^a^We do not have the demographics of two doctors. ^b^These doctors had experience and knowledge about primary care and discussions with them helped to illustrate differences in work roles between peripheral and higher levels of the public system.

We used an unstructured topic guide to facilitate our discussions with the doctors (see Table 1 for key themes in the topic guide). During these discussions, doctors were encouraged to talk about various situations that came up during their day-to-day work and the ways through which they dealt with them. These concrete examples were used as anchors to understand and delve deeper into factors that shaped doctors’ attitudes and actions.

Most interviews lasted about an hour and were conducted in a mix of English and the local language.The interviews and visits were done between April and September 2018 in four iterations. We voice-recorded 15 interviews, whilst also taking some notes, but judged that we would be able to get less-guarded responses if we did not record. Hence, the latter half of the interviews were semi-recorded or unrecorded; accompanied, rather, by lengthy notes written during and post the interview. Many doctors also spent time with the first author showing the different services available in the health center; this time provided an opportunity for informal conversations that yielded rich information. Most of the interviews were done by the first author. Logistical support for data collection was provided by the Center for Social Medicine, Pravara Institute of Medical Sciences.

### Data Analysis

All recorded interviews were transcribed into English. The transcripts and field notes were first open-coded. The initial themes that emerged (such as the categories of influences, or decisions doctors took) appeared to resonate well with the theoretical lens of SLB, so we used concepts from this theory to deepen the analysis. We worked iteratively with the list of themes that emerged and constructed data displays (tables and charts) to organize and detect patterns of relationships in the data. We used NVivo version 12 to aid the coding process. The first author led the analysis, but the preliminary analytical summaries and data displays were shared with the other authors to allow for interpretation. During the last iteration of data collection, these summaries were shared with 2 additional doctors from the public system for further validation.

Consent from all participants was taken; but ten doctors preferred to give verbal rather than written consent for the interviews (the doctors were willing to participate but did not want to sign a form). When given permission to record, interviews were audio-recorded. All recordings were deleted after being transcribed. All data in the transcripts has been anonymised. In the paper, we have not mentioned the name of the rural area or of the particular health centers so as to protect the identity of health system staff. Permissions for the study were granted by the state of Maharashtra as well as district-level authorities.

### Use of the Street Level Bureaucracy Theory for Analysis

Lipsky’s original theory- set in contexts where frontline workers were blamed for being aggressive and irresponsive to client needs- contended that frontline workers exhibited these behaviours as responses to challenges in the public sector bureaucracy.^1^ These reactions were, thus, not “*random acts of noncompliant behaviour,*”^5^ but ways through which the doctors used their discretionary power and ‘coped.’^30^ Lipksy originally categorised the coping behaviours of frontline workers as those pertaining to modifying client demands (thus maximising use of resources), modifying job objectives (including mentally withdrawing from jobs) and modifying perceptions of clients (by favouring some instead of all).^1^ Subsequent literature has tried to expand on these categories. For instance, Brehm and Gates categorised the behaviours of bureaucrats into three categories – ‘working’ (devoting extra-ordinary effort, more than what is obligatory), ‘sabotaging’ (deliberately working against policy goals) and ‘shirking’ (putting less effort into policy goals or more efforts into non-policy goals).^31^ More recently, Tummers et al had put forth a typology of coping behaviours, synthesized from literature across several disciplines. Here, coping was defined as “*the cognitive and behavioural efforts made by frontline workers to master, tolerate, or reduce external and internal demands and conflicts among them during policy implementation.”*^32^ We found this typology to be comprehensive; and the categorization of coping behaviours used here seemed to fit well with the initial analytical themes that we had inductively extracted. We have adapted this typology for this study and have looked at coping in terms of:

1. *specific instances of coping*– specific examples of action observed or reported by doctors (like a doctor not doing an out-patient clinic that is mandated)
2. *coping strategies* – mechanisms that surround coping instances (like a doctor rationing and routinising care) and
3. *adaptive processes* – broader attitudes and behaviours that have become routine among doctors in the public primary care sector (like doctors becoming risk-averse or learning to game the system).

Tummers et al had also grouped coping behaviours as those that moved towards clients (made pragmatic adjustments to aid client needs), those that moved away from clients (actions that avoided meaningful interactions that can benefit clients) and those that moved against clients (being confrontational with clients).^32^ We could not find instances of coping behaviours that moved against clients in our data, so we have used only the other two groups to report our findings.

After delineating the nature of coping behaviours by doctors, we also examined why such behaviours persisted in the frontlines of the public health system. Lipsky included constraints of resources, ambiguous policies, increased work burden and work stress as some of the issues encountered at the frontline.^1^ Derived from these ideas, many efforts have been made to systematically understand factors that influenced street-level action. Previous literature from LMICs has categorised factors shaping coping behaviours of street level bureaucrats as: individual characteristics (values, norms, gender, profession); organizational characteristics (structure, formal and informal rules, culture, workload); community or patient characteristics (needs, perceptions of workers); and other broader socio-political systems.^2-4^ We have used similar categories to examine our findings. We have also looked specifically at the interactions between doctors’ professional identities and issues in the organizational context- for these interactions played out strongly in our data.

## Results

We have reported the findings of this paper in two sections. First, we have described various coping attitudes and behaviours exhibited by doctors in primary health centers (section 1). Then, we have examined why such coping behaviours persist in health system, thereby contributing to gaps in service delivery at primary health centers (section 2).

### Section 1: The Nature of Coping Behaviours Reported by Doctors in Primary Health Centers

Below, we have summarised a list of coping behaviours found in our data, and have defined these behaviours using adaptations of definitions used previously in SLB literature.^1,3,7,32^ Adapting from Tummers et al classification,^32^ we have discussed coping behaviours in terms of “*moving away from patients*” (doing actions/interactions that are less meaningful to the community) and “*moving towards patients”(*making practical adjustments to benefit the community) (see Table 2).

**Table 2:** A Summary of Coping Behaviours at Primary Health Centers

	**Coping Strategies**	**Adaptive Processes**
Moving away from patients	*Rationing care:*Doctors rationed care by decreasing service availability or decreasing attractiveness of service and hence save resources in a constrained environment. *Routinizing care:* Doctors routinized care by doing a less thorough, non- systematic processing of patients. *Patient categorization:*Doctors selected some patients in lieu of others and have informal selection criteria and barriers. *Invoking a different policy understanding:* Doctors invoked a meaning of the policy that was different from what was intended thereby reduce commitment to intended goals. *Priority-setting:* Doctors worked only on what they perceived as priorities, especially those encouraged within the organization. *Distancing:* Doctors remained mentally withdrawn from patients/beneficiaries and are non-responsive to their problems.	Became risk-averse, sacrificed the professional and learnt the “rules of the game,” gaming the system. Overall policy alienation.
Moving towards patients	*Rule bending:*Doctors adjusted a formal rule as per patient demands. *Retaining professional identities:* Within available circumstances, doctors tried to be better professionals and serve the community.	Doctors tried to be better professionals even while being risk-averse.

#### Coping: Moving Away From Patients

Our discussions with doctors revealed several coping behaviours at primary health centers that moved away from patients (Table 3 highlights some reported experiences). One of the key roles assigned to doctors at primary health centers was to provide basic outpatient care to patients and refer them for continued treatment to the higher tiers of the system if needed. However, doctors often did not do justice to this role. Indeed, many doctors resorted to conducting only one Out Patient Clinic in a day despite being mandated to conduct two (see Table 3, action 1). Doctors reported that they often saw patients quickly and did not spend time engaging deeply with them (see Table 3, actions 2- 5). Some doctors also reported instances where they were forced to give priority to certain patients (who did not need medical priority) due to the demands of local politicians (see Table 3, action 4). Some of them acknowledged that they referred more patients to the higher tiers of the system than clinically necessary (see Table 3, action 6). In short, most doctors appeared to deliver rationed and routinized outpatient care, and often distanced themselves from patients’ needs.

**Table 3:** Doctors’ Coping Behaviours That Moved Away From Patients/Beneficiaries

	**Illustrative Instances of Coping**	**Coping Strategies**	**Adaptive Processes**	**Implications on Care Provided**
Outpatient care	One doctor reported having adequate drug supplies to do only one outpatient clinic in a day, rather than the mandated two (Action 1).	Rationing care	Resignation Sacrificing the professional Risk-averseness	**Implications for outpatient care** Doctors conducted only one outpatient clinic in a day (despite being mandated to conduct two). Doctors saw patients very quickly and made professional compromises while providing care. Doctors did not provide equal care to all patients. Doctors referred more patients than clinically required.
	One doctor reported that he saw the medicines that he had given a patient thrown on the footpath in front of the health facility. This incident made him feel that it was no use trying to help patients who did not trust his professional opinion. Now, the doctor resorts to judging patients instinctively and takes time/effort only if the patient appeared amenable (Action 2).	Routinising care Patient categorization		
	Many doctors shared that the ‘actual’ work of the primary health center was to implement programs and schemes; and outpatient clinics were not important part of their reporting mandates. Hence, they rapidly dealt with outpatient work and focussed on other issues. It was felt that outpatient work was neither appreciated by patients or the organization (Action 3).	Prioritizing Routinizing care		
	Doctors often reported giving preferential treatment to friends and relatives of local politicians. If they refused to do so, there was danger of these politicians creating obstacles to other outreach work (Action 4).	Patient categorization		
	Some doctors reported that they had only a few drugs to work with in the health center, so they prescribed the same drugs again and again to patients (even while knowing that these drugs were not the best clinical choices). These doctors reported that patients would get angry if they sent them back without drugs or asked them to buy drugs from outside. At the same time, they did not have freedom within the institution to get better drugs. So, they resorted to giving drugs perfunctorily (Action 5).	Routinizing care Distancing		
	One doctor referred all cases of delivery that came to his health center- since he felt that he neither had staff or facilities to deal with emergencies. He did not want to take a ‘risk’ (Action 6).	Invoking different policy understanding		
Programs	Many doctors shared that too many schemes ran from the health centers; and staff numbers were adequate to do all outreach work. Hence, they overlooked short-cuts taken by staff during outreach (Action 7).	Rationing Routinizing Distancing	Policy alienation Gaming the system Resignation Risk-averseness.	**Implications on programs** Doctors ignored shortcuts taken by staff, contributing to compromises in outreach. Doctors reconciled themselves to the diluted, perfunctory delivery of schemes.
	One doctor tried to take action against a nurse who refused to complete duty-hours, but he received no support from the authorities to suspend her. He was told to “adjust” and carry on. After this incident, he stopped trying to better the implementation of schemes (Action 8).	Routinizing Distancing		
	One doctor was told to open bank accounts for all patients with respect to a health scheme. He felt he should be given only “technical work” and not work of this sort, so he monitored only a few account openings (Action 9).	Invoking different policy understanding		
	A doctor once forgot to call a local politician for an inauguration event of an immunization campaign, and this led to several implementation obstacles. Post this incident, he felt that politically appropriate launches were more important than the technicalities of the campaign itself- and hence changed the focus of his work (Action 10).	Invoking different policy understanding		

Another important role of doctors in primary health centers was to administer and manage several vertically-run programs and schemes that ran from these facilities. Most of these programs involved a community outreach component that was to be done by support staff, under the overall supervision of doctors. Doctors often shared that they had little interest in the administrative work that surrounded the implementation of these programs; but had no choice about such work. Many doctors reported resorting to short-cuts (or ignoring short-cuts taken by other staff) in implementing these programs (see Table 3, action 7-10). They often routinized program implementation and invoked a different meaning of the program in order to justify the short-cuts routinely taken (see Table 3, action 9-10).

Over time, most doctors in primary health facilities reported getting used to a way of functioning that was less than ideal. They became resigned and accepted their inability to change things for the better. This attitude of resignation was visible in many conversations with doctors:

“*I have a colleague. After years in this system, nothing bothers him, he has become rough and tough and does little work. Hardly comes to the center. He has started feeling ‘nothing will happen to us-then why should we work?’ We have stopped caring about what people want”*(Medical Officer, Male, 8 years of work experience).

“*But then this is way the system is. Nothing works. Who will make things better? Why? Best to keep quiet and work with what the government gives”* (Medical Officer, Male, 20 years of work experience).

Further, most doctors got used to taking risk-averse decisions in primary health centers, as well as making professional compromises in the day-to-day work (see Table 3, column on adaptive strategies across actions 1-10). They felt alienated from the programs and schemes that ran from primary health centers, did not find meaning in implementing them well, and sometimes resorted to gaming the system (see Table 3, column on adaptive strategies across actions 6-10). Most doctors at primary health centers today seemed to have reconciled themselves to the idea that survival in the government system meant the sacrifice of their “*clinician”* hats. As one doctor put it, the work in primary health centers had become about

*“Work as usual. Not do too much extra. Just do what’s possible”* (Medical Officer, Male, 13 years of experience).

What were the implications of doctors’ coping behaviours on the work of primary health centers? The resigned attitudes of doctors, their distancing from patients, the rationing and routinising of care along with the adoption of a risk-averse approach contributed to the delivery of poor-quality outpatient care. Further, the same attitudes of resignation and inability to change things for the better contributed to the perfunctory and diluted implementation of programs at primary health centers.

#### Coping: Moving Towards Patients

We found only a few doctors who reported behaviours that moved towards patients. These doctors bent rules for the sake of patients rather than for personal benefit and tried to retain their professional identities (see Table 4, actions 1-3). It was interesting to note that even while these doctors bent rules to help patients, these rules were often bent in *“safe mode*.” For instance, in Table 4 action 1, the doctor who bent rules by writing prescriptions for patients when drugs were not available, did not do so for all patients; he only did so when he felt that a particular patient would accept the act. In other circumstances, he routinized care as usual.

**Table 4:** Doctors’ Coping Behaviours That Moved Towards Patients

**Illustrative Instances of Coping**	**Coping Strategies**	**Coping - Adaptive Processes**	**Implications on Care Provision**
A few doctors acknowledged writing informal prescriptions to help patients when appropriate drugs were not available at the health centers. These doctors did this only when they felt confident that these patients would not complain about this action (Action 1).	Rule bending	Doctors felt conflicted. They attempted to retain a professional identity within a risk-averse frame of working	Some attempts being made to deliver primary care in line with professional ideals.
One doctor felt that the local private practitioner fleeced poor rural patients of their hard-earned money. So, he tried to make his outpatient clinic more attractive to patients by behaving the way private practitioners did- by smiling at patients, giving instant relief treatments sought by patients and incorporating “drama” into his daily clinic (Action 2).	Retaining professional identities		
One doctor refused to provide obstetric care at his primary health center despite top-down pressure to do so. He felt that he would be risking the life of the patient by doing so- and shared that he would rather face the anger of his superiors than put women’s lives at risk. However, he tried to follow-up on many of his referrals (Action 3).	Rule-bending Constructing a professional identity		

### Section 2: Factors That Shaped the Coping Behaviours of Doctors: Professional Identities Versus Organisational Constraints

Why did doctors in primary health centers behave the way they did? The coping instances described in Tables 3 and 4 provide a glimpse of the reasons given by doctors for their actions; however, in this section, we have analysed these reasons further. Table 5 summarizes the factors that shaped doctors’ actions.

**Table 5:** Factors That Influenced Doctors’ Attitudes and Actions at Primary Health Centers

**Label**	** Details**	**Underlying Beliefs**
Profession-related factors	The primary health center was not perceived as a place for good clinical work- since it had few drugs and equipment to work with. Primary care roles were perceived as hindering professional growth. Doctors’ roles in primary health centers was perceived as reduced to being that of administrators and social workers, leading to a lack of professional satisfaction. Doctors felt professionally isolated in primary health centers.	Perceived lack of professional value in executing primary care roles. Did not want to work in public systems; preferred the private sector. Public sector jobs needed one to be risk-averse. Mistrust and lack of connect with rural patients. Other personal values.
Organizational factors	*Perceived structural deterrents at primary health centers* Limited facilities and drugs, poor infrastructure. Vertical programs that functioned with strong targets. Lack of adequate support staff for outreach. *Non-structural organizational issues* “Narrow” mandates to provide only certain services. Too many targets were imposed by higher-ups. Emphasis on reporting rather than doing “good” work. Some doctors felt that they had little actual authority over outreach staff-even as heads of these centers. Doctors had low confidence that authorities in the system would support them in case of mishaps.	
Other socio-political factors	Doctors felt they could do little for patients due to the constraints of drugs and equipment they faced. Doctors reported facing clinically irrational demands from patients. Local politicians demanded preferential services. Reports of violence against doctors engendered fear.	

The factors in Table 5 were separated from each other for the purpose of examination; but it was the interactions between these factors that were interesting. Particularly interesting were the interactions between doctors’ twin identities as a clinician (professional factors) and a bureaucrat in the system (organizational factors). Clearly, there was a mismatch between the professional goals of doctors and the roles they were assigned at primary health centers. Discussions with doctors revealed that the provision of routine outpatient care for basic ailments like cough, cold and fever was not perceived as clinically exciting. Doctors wanted “good” clinical work, that is, cases where the diagnosis was difficult, and where their professional expertise added value. The work at primary health centers made them feel cheated of opportunities to use their professional knowledge. Such work was often defined by doctors as *“not doing much,” “nothing big*” and “*nothing more than first aid and paper-work*” – and had a derisive tone attached to it. Doctors felt that their work in primary health centers had mainly become that of a “*social worker*” and an “*administrator”* rather than that of a “*real doctor.”*

The loss of doctors’ professional identity at primary health facilities was not only linked to mismatched roles, but also to poor relationships with the community. While all doctors reported getting into the medical profession to “*serve the community*,” most acknowledged becoming disillusioned about this goal during the course of their work. Doctors shared that patients often came into primary health centers with low expectations of care, only when they had run out of alternatives, and did not ‘trust’ the medicines provided. Many doctors reported that they feared being subjected to physical violence by community members in case of a clinical mishap. Hence, they did not attempt potentially complex care (like a complicated delivery) even when the situation demanded it; and often took risk-averse decisions. They referred more cases than clinically necessary – for doctors shared that a clinical mishap “*gave the government a bad name*,” following which they would be blamed for their actions not only by the patients, but also by the higher authorities. Disillusioned by their lack of support for being ‘good’ professionals, doctors felt helpless and unable to help the community. Hence, they often stopped trying to live up to the professional standards they had once recognised for themselves. In the course of doing so, they sacrificed their clinician hats. For most doctors, this was a great burden to bear (see Box 2 for illustrative quotes on this point).

**Box 2. The Sacrifice of the Clinician’s Hat And Being “Just a” Bureaucrat: Illustrative Quotes**

*“This work is for people who are administratively strong. Last week, I had to open bank accounts for all people and get signatures. Not really the work I want to do”*(Medical Officer, Male, 4 years of work experience in primary care and 4 years in higher tiers).

*“Some instruction will come from above…people from the top pick up the phone and say ‘send this report it, that report’ that all has to be done at (name of the center). Nothing that uses my doctor skills”*(Medical Officer, Male, 1 year of work experience).

*“Down in the system, people create trouble, politicians create trouble and you can’t work. I so much prefer the (district level) hospital”*(Female doctor, who used to work at primary levels previously).

*“Sometimes I feel really bad…with so much difficulty I have obtained this qualification…this big degree…and after this, what kind of service I am giving people? No medicines, no investigations…I don’t even have a IV line…why will a patient come to me? What can I do for them?”*(Medical Officer, Male, 13 years of work experience).

*“What change? Better to let things be as they are…how things are going on, let them go on the same way. Nothing is in our hands, right? See, like today, we have given a demand for drugs, I know nothing will come…still I demand…and we adjust with what is there”*(Medical Officer, Male, 9 years of work experience).

However, we also encountered a few doctors in the system who had managed to retain vestiges of their professional identity and tried to ‘cope’ with the situation by adopting positive behaviours (for instance, see Box 3. This doctor tried hard not to become just a “*paper-work*” person). But such doctors were few, and most doctors in the public system appeared to have accepted their diminished professional status.

**Box 3. Case Illustrating a Doctor Who Tried to Retain His Professional Identity by Adopting Positive Behaviours**

This doctor, having 10 years of experience shared that he had invested too much in being a “professional”. Hence, he did not want to limit himself to doing only the minimal administrative work requirements of the health center.

However, he also felt that there was little appreciation for the good clinical care practices that he had been trying to bring into the health center. Since his posting, attendance at the outpatient clinic at the health center had tripled, he said, but this fact was not even acknowledged by his superiors. This doctor had also tried to improve outreach services at the health center- by trying to push his outreach team to do deeper work. He admitted his inability to do so; for while he changed his routine and stayed at the primary health center during his entire official duty hours, he could not motivate his staff to do so. The doctor also said that most of his staff was older, had been in the government system for more than 20 years; and did not always listen to him.

The doctor reported being wary of getting institutionalised. He was worried that over time, he would get habituated to such a system of working and he actively resisted getting into what he called as *“the typical government mindset of not doing anything more.”*However, he also defended the government system saying they were not entirely at fault; in the past, the media had often exaggerated issues and dramatized mishaps and accidents and hence, public systems had learnt to be careful.

This doctor understood the “rules” of the game, but he did not want to get reduced to becoming a “*paper-work*” person. Hence, he tried to be a better professional within the broad framework of being risk-averse.

Interestingly, even doctors who had accepted the dominance of their bureaucratic identity over the professional one did not always do justice to the ‘administrative’ roles assigned to them. Doctors reported several constraints at primary health facilities that prevented them from being good administrators (see Box 4 for quotes). They shared being overwhelmed by the targets and the number of schemes/programs imposed on them by district-level authorities. They shared that schemes and programs often ‘came and went’ and doctors were often not given explanations for why a particular scheme was withdrawn suddenly. Further, doctors also shared that the numbers of support staff at primary health centers were inadequate to do the kind of work that was expected from the health center by higher-level authorities. Thus, most of them felt that they had no option but to ignore the short-cuts taken by support staff at the health centers while executing program-related work. They often ended up executing programs mainly for ‘*reporting purposes.’*

**Box 4. Challenges Faced in the Organizational Set up by Doctors: Illustrative Quotes**

*“…Day by day., the national programs burden is increasing, and new schemes are being launched…the field staff finds it tough…what will they do? Reporting or field work? Or administration…all this affects the quality of work”*(Medical Officer, Male, 12 years of work experience).

*“There are so many lacunae in government…so what can we do, you tell me? Medicines are not there…equipment is not there…we can do nothing. We are like you only…we can do nothing. It’s a bigger pattern outside of our control…”*(Medical Officer, Male, 20 years of work experience).

*“The issue is that once you get into the government, there is a danger of you getting institutionalised. The more time you spend in certain settings, you become like that…one has to be very careful not to fall into the rut of doing nothing”*(Medical Officer, Male, 10 years of work experience).

*“It is all government policy to make which policy, which scheme…and how to implement it…it has nothing to go with me…I just follow…I do my work”*(Medical Officer, female, <1 year of work experience).

Doctors also shared that doing anything “extra” while managing programs (that was beyond their reporting mandates) posed a “risk;” and such actions, though not formally prohibited, were not encouraged or supported in the public system. A few doctors reported failed attempts to improve this state of affairs. One doctor had tried to send a memo to district authorities for the suspension of a support staff member who had refused to do outreach work; but there was no action taken on his memo despite many reminders. That experience had taught him that his job was to ‘*make do with what he had’* and not to improve the implementation of programs. In fact, we encountered the idea that it was not the doctor’s job to improve implementation processes, many times during our conversations. Box 5 illustrates a detailed case of this attitude pertaining to a screening program that was launched during the time of our discussions.

**Box 5. Experiences of Doctors Who Participated in a Screening Program**

During the time of our discussions, doctors were asked to participate in a massive screening campaign for non-communicable diseases (NCDs); but no treatment was available post-diagnosis for patients as per policy. Many doctors felt that screening without providing drugs was unethical; some shared that they were not trained to screen; others said that the diagnosis of NCDs was beyond the scope of primary-level care. However, these implementation concerns were rarely discussed with higher-level managers. It was generally acknowledged that top-level administrators were ‘*happy’* as long as some version of the NCD policy directive was getting implemented on the ground. One doctor said that improving implementation processes was subtly discouraged in the public system and he had learnt through experience that it was *“not his business*” to improve implementation. Most doctors had worked out a way to implement the scheme for “name-sake” only- that is, superficially, by completing minimally required screening targets.

Younger doctors sometimes shared that they felt uncomfortable not doing anything about the routine compromises they had to made during their work at primary health centers. However, they had been *‘taught’*by the more senior doctors that making efforts to change the way things worked in the public system was essentially pointless. More experienced doctors shared they had become used to this work and hence felt little distress when they compromised in care provision; for they had accepted these issues as an integral part of the job.

Below, we summarise the study findings from section 1 and 2. Doctors reported several challenges in delivering primary healthcare-including the lack of resources, the top-down imposition of programs that were not meaningful to them, limited support from the organization to improve processes as well as professional disinterest. These challenges contributed to the adoption of a range of coping attitudes and behaviours. Most doctors routinized and rationed care, and often became resigned and risk-averse. Most of them felt a deep loss of their professional identity, and accepted this loss as an inevitable part of a public sector job. Further, most doctors felt that they could not do justice to the administrative roles that they had been assigned. Such coping behaviours, on balance are likely to impact negatively on the quality of care provided from primary health centers.

## Discussion

While the volume of actor-centric, empirical studies in the field of policy implementation has been growing recently in LMICs, few studies have looked at explicitly at doctors - as Street Level Bureaucrats. One reason for this could be that doctors have typically been considered a group of highly-skilled professionals with inherent discretionary power^16^; allowing their bureaucratic identity to be subsumed into their professional one. Our study shows, however, that there is merit in looking at doctors’ actions using SLB theory. Through the SLB lens, doctors’ actions are not treated solely as professional decisions. Instead, the actions are seen as ways through which these professionals ‘cope’ with a wide range of contextual factors; and ‘coping’ causes them to sustain particular attitudes and work-routines in the long-run. Thus, the use of the SLB lens expands the understanding of doctors’ actions as contextually situated routines.

‘Coping’ has always been a central concept in SLB theory,^1-3^ and some empirical LMIC literature does touch on how frontline health staff ‘cope’ by adopting certain attitudes and actions (such as rationing, distancing from patients and categorising patients) during policy implementation.^7,9,10,12,15^ However, the knowledge base is still limited and more studies that systematically categorise coping behaviours and elicit nuanced accounts of these behaviours are needed from LMIC health contexts. In this study, the consideration of coping at various levels- as specific instances, as strategies that surround these instances, and also as aggregated adaptive processes (adapted from Tummers and colleagues’ framework^32^) – has enabled a systematic and detailed understanding of doctors’ coping behaviours in primary healthcare settings. To elaborate, examining individual coping instances helped to dissect doctors’ actions and these instances have served as illustrative ideas around which to deepen discussion. Coping strategies that are more abstract than instances (such as routinising, rationing and categorising patients) have also helped to understand the mechanisms that define coping instances. Broader adaptive processes clarified how certain patterns of attitudes and behaviours eventually become part of work-routines at primary health centers. Overall, examining coping through these three levels has added depth as well as clarity to our understanding of doctors’ actions. Our analysis suggests the usefulness of further empirical work from LMICs that similarly considers coping behaviours in this layered way.

In this study, we have paid particular attention to the broader adaptive processes of coping- resignation, risk-averseness and the sacrifice of professional ideals. This is because such processes present a window for understanding informal routines – the “*practical norms*”^6^ – that develop over time and persist in public sector organizations. Our findings indicate that these adaptive processes are not specific to any particular scheme or policy; rather, they underlie all activities implemented from primary health centers and form the basis for *de facto* policy. Other studies have also touched upon such adaptive behaviours. Health providers in public health systems have expressed concerns about being used as *“scapegoats”* by the system,^13^ have reported being scared of being subjected to censure by the media and the community^33^; and also have expressed feeling *“pursued rather than protected in the exercise of medical care;”*^11^ all of which contributes to the risk-averse coping routines that persist in public health systems.

In our study, adaptive coping processes often seemed to form the basis for the “*collective divergence”*^34^ of doctors from intended policy goals. Over time, most doctors seem to have learnt that survival in the system implies working mainly with formally imposed and heavily monitored policy mandates (ie, vertically-funded health programmes) and ignoring other services; and, as a result, have, over time, “*drifted towards compatibility with the way (things are) evaluated”* in the public sector.^1^ However, it is important to note that these imposed policy mandates appear only to influence “what” must be done at primary health centers, rather than the more nuanced “how” of the tasks involved- which is often, inevitably, left to the discretion of doctors. Thus, in a risk-averse environment with resigned attitudes and low levels of professional enthusiasm, policy mandates are often carried out in perfunctory ways. There is little ownership of tasks beyond obligatory reporting requirements; and as Stone^35^ puts it, there always seems to exist a *“felt need to go on to the next client, the next report, or the next item of business.”*Even exceptional providers who try hard to take ownership of tasks are limited by resources, lack of organizational support, and the constant informal pressure to be risk-averse; and these limitations often leaves them frustrated. These experiences result in the perfunctory and diluted implementation of primary healthcare policies.

This study also highlights a range of factors as influencing doctors’ practices in Indian primary health centers. We have categorised these as the underlying beliefs of doctors (values, individual beliefs and personal goals), professional (the lack of professional interest in primary care roles, missed opportunities to use clinical expertise and professional isolation), organizational (risk-averseness, strongly imposed top-down mandates and infrastructural constraints) and local socio-political (lack of community engagement and local political interference) factors. Similar categorizations of influencing factors have been used by others as well.^2,3^ Many of the organizational and local socio-political factors elicited in this study – resource constraints, structural deficits, issues with organizational culture and relationships, and lack of connect with the community - have been noted in other empirical studies on frontline policy implementation that involve doctors,^13,14^ as well as other cadres of frontline staff.^7-12,36-38^

In our findings, the set of ‘professional’ factors play out strongly, and these interact in various ways with ‘organizational’ factors to shape doctors’ actions. Although one other study has noted these interactions, as well as the challenges that doctors face in dealing with the duality of being a professional (a clinician) as well as a bureaucrat,^14^ most SLB studies underplay the influence of professional factors over frontline worker actions.^39^ Lipsky in his original work does refer to professional identities as playing a role in shaping frontline worker response, but studies from the LMIC health sector that use SLB theory have usually placed less emphasis on this aspect. Our findings underscore the importance of examining both roles (being a ‘professional’ as well as being a ‘bureaucrat’) when considering frontline cadres (like doctors) that have a strong sense of professional identity. In our study, we found that most doctors felt they could not do justice to either of these roles due to the many organizational constraints they faced. This has led to long-term demotivation and inculcated the attitudes of resignation among doctors that seem to have contributed to the delivery of poor quality care.

Some limitations of this study are noted. First, it was not easy to collect data on street-level coping behaviours. While doctors were often willing to talk about general challenges faced during work in the public system, it was not always easy for doctors to talk about specific instances. Some doctors preferred to speak in third person (about difficult situations faced by a colleague) rather than about themselves. Conversations that were not tape-recorded in general yielded better information, especially on certain themes pertaining to the public sector organization. However, even during untaped conversations, there were certain themes- corruption, taking and giving of bribes by doctors and private practice - known to exist in similar settings,^40-41^ that were not fully discussed by study participants, and so have not been reported in this paper. One important point that aided frank conversations was the fact that the first author who conducted the interviews was a doctoral student. A letter from the university seemed to assure doctors that they were not being tested for their competence or being evaluated by the organization secretly. Although we observed doctors while waiting at primary health centers, the evidence presented in this paper is mostly self-reported actions which could not be triangulated with observations. Finally, although we did purposefully select female doctor respondents, the majority of doctors available in these rural regions are men and so the opinions we report are male-dominated.

This study was done in one rural area, and we do not claim that the experiences we have reported here are representative of all doctors in rural primary healthcare settings. However, the rich, in-depth data from our study offers interesting insights into the nature of coping behaviours in such settings and factors that shape these behaviours. Based on these insights, we reflect below on what the study findings could mean for primary healthcare policies in India and other similar LMIC health contexts.

Policies in India have historically been concerned about the challenge of recruiting doctors for work at primary health centers; as many doctors prefer to opt for private practice.^42,43^ Even among those who work at primary health centers, frustration and lack of motivation has been documented.^44,45^ However, there have been few interventions in the public sector in India to remedy the situation, besides technical training and structured incentives. Our evidence clearly points to the need for a different set of interventions; those that can deal with the fear of blame and frustration, help deal with challenging relationships and institute oversight mechanisms beyond measurement of specific performance indicators.

While the exact nature of these interventions needs to be context-specific, the general principles that surround a set of interventions to improve provider behaviour would likely remain the same across resource-constrained settings. Other LMIC studies using the SLB lens have pointed out the need for interventions that deal with ‘*advocacy, awareness-raising, and debate at the frontline,’*^9^ thereby making policies more meaningful to health providers.^7,9,11^ Interventions suggested in the literature from other LMICs include values’ clarification workshops, supportive supervision, engaging staff in reflection, consulting with staff regarding policy changes rather than imposing them top-down, using appreciative enquiry techniques and generally strengthening leadership at the frontline.^2,7,46-49^ The need to create spaces for deliberative and reflective practice has been pointed out.^2^ Recognising informal practices among frontline health workers and bringing about changes in the organizational culture that allow frontline health providers better access to and control over resources has also been shown as important.^50,51^ In summary, the need for ‘*new and more creative strategies’*for motivating healthcare workers has been emphasized.^52^

To conclude, this study attempts to adds to the policy implementation literature in LMICs that applies the SLB lens. It illustrates that doctors’ decisions at primary health centers are shaped by their professional values as well as by formal and informal organizational requirements. It highlights that a range of informal practices have become ‘routinised’ in the health system, and that these can contribute to the disjuncture between policy goals and frontline realities. Thus, this study confirms the usefulness of the SLB lens in studying routine health policy implementation in LMICs.

## Acknowledgements

This paper has been funded through the Health Policy Analysis Fellowship programme, supported by the Alliance for Health Policy and Systems Research, Switzerland. We acknowledge the mentor team of the fellowship program-Maylene Shung-king, Irene Agyepong, Jeremy Shiffman and Zubin Shroff; and all the fellows of the program for their reviews and comments on this work. We thank the Center for Social Medicine, Pravara Institute of Medical Sciences, in Ahmad Nagar, Maharashtra, for logistical support during data collection- in particular, the interns who worked with us and faculty of the school, Dr. Somasundaram and Dr. Thitame. We thank the health staff who shared their valuable thoughts with us during the study. We particularly thank Professor T. Sundararaman, Independent consultant and former dean, School of Health Systems Studies, Tata Institute of Social Sciences, Mumbai and Professor Surinder Jaswal, Deputy Director (Research), Tata Institute of Social Sciences, Mumbai, for their inputs on the results of this study.

## Ethical issues

Ethical approval for the study was taken from the Institutional Review Board at the Tata Institute of Social Sciences, Mumbai, India- in April 2018.

## Competing interests

Authors declare that they have no competing interests.

## Authors’ contributions

SR designed this part of the study, with mentorship from LG and SM. Data collection was done by SR, and LG, SM, and NG commented on the analysis. The first draft of the paper was written by SR, and reviewed by LG, SM, and NG. The final draft of this paper has been reviewed by all four authors. SR is the guarantor of the paper.

## Authors’ affiliations

^1^School of Health Systems Studies, Tata Institute of Social Sciences, Mumbai, India. ^2^Division of Health Policy and Systems, University of Cape Town, Cape Town, South Africa. ^3^Department of Global Health and Development, London School of Hygiene and Tropical Medicine, London, UK. ^4^Center for Health and Social Sciences, School of Health Systems Studies, Tata Institute of Social Sciences, Mumbai, India.

## Key Messages

**Implications for policy makers**

- This study contributes to empirical literature from low- and middle-income countries (LMICs) that explains how the attitudes and behaviours of frontline health providers shape policies.
- In such settings, doctors have often been blamed for not implementing policies well and for not living up to professional expectations. But an alternate way of looking at these actions is by considering these as ways in which doctors ‘cope’ with the challenging situations they face during day-to-day work.
- Doctors face several constraints in the implementation of primary care – lack of professional interest in primary care roles, top-down policy mandates imposed on them, lack of community trust and systemically-engendered risk aversion. These constraints lead to demotivation, resignation and a loss of professional identity among doctors. Ultimately, they deliver a compromised version of primary care, different from that of policy expectations.
- Further technical training of doctors has often been offered as a means of improving the delivery of primary care in public health settings in LMICs. This study suggests that other interventions - such as supportive supervision, the use of appreciative enquiry techniques, as well as consultations and deliberation with staff regarding policy implementation-would also be important to improve care.

**Implications for public**

In this study, we look at how health providers’ attitudes and behaviours shape policies, through the example of doctors who work in the public, primary health sector in India. Doctors reported that they had limited professional interest in delivering primary care, and were constrained by other issues such as the lack of infrastructure, the imposition of too many targets and a lack of connection with the community. Doctors’ also acknowledged that such constraints led them to adopt behaviours that could potentially comprise the delivery of primary care. These findings suggest that the provision of technical training or the mere imposition of top-down targets on health providers are not adequate to make policies succeed. To ensure policy success, people in the frontline have to be supported and motivated in other ways.

## References

[1] Lipsky M. Street-Level Bureaucracy: Dilemmas of the Individual in Public Services. New York: Russell Sage Foundation; 1980.

[2] Gilson L. Lipsky’s street level bureaucracy. In: Page E, Lodge M, Balla S, eds. Oxford Handbook of the Classics of Public Policy. Oxford: Oxford University Press; 2015.

[3] Erasmus E (2014). The use of street-level bureaucracy theory in health policy analysis in low- and middle-income countries: a meta-ethnographic synthesis. Health Policy Plan.

[4] Rice D (2013). Street-level bureaucrats and the welfare state: toward a micro-institutionalist theory of policy implementation. Adm Soc.

[5] Brodkin EZ (2012). Reflections on street-level bureaucracy: past, present, and future. Public Adm Rev.

[6] Olivier de Sardan JP, Diarra A, Moha M (2017). Travelling models and the challenge of pragmatic contexts and practical norms: the case of maternal health. Health Res Policy Syst.

[7] Walker L, Gilson L (2004). ‘We are bitter but we are satisfied’: nurses as street-level bureaucrats in South Africa. Soc Sci Med.

[8] Scott V, Mathews V, Gilson L (2012). Constraints to implementing an equity-promoting staff allocation policy: understanding mid-level managers’ and nurses’ perspectives affecting implementation in South Africa. Health Policy Plan.

[9] Diarra A, Ousseini A (2015). The coping strategies of front-line health workers in the context of user fee exemptions in Niger. BMC Health Serv Res.

[10] Atinga RA, Agyepong IA, Esena RK (2018). Ghana’s community-based primary health care: why women and children are ‘disadvantaged’ by its implementation. Soc Sci Med.

[11] Van der Veken K, Dkhimi F, Marchal B, Decat P (2018). “They are after quantity, not quality”: health providers’ perceptions of fee exemption policies in Morocco. Int J Health Policy Manag.

[12] Nunes J, Lotta G (2019). Discretion, power and the reproduction of inequality in health policy implementation: practices, discursive styles and classifications of Brazil’s community health workers. Soc Sci Med.

[13] Karadaghi G, Willott C (2015). Doctors as the governing body of the Kurdish health system: exploring upward and downward accountability among physicians and its influence on the adoption of coping behaviours. Hum Resour Health.

[14] Gaede BM (2016). Doctors as street-level bureaucrats in a rural hospital in South Africa. Rural Remote Health.

[15] Kelly G. Ways of Coping: How Medical Doctors Manage their Work within the Social Security System in South Africa. Working Paper. Centre for Social Science Research, University of Cape Town; 2017.

[16] Gaede BM. Civil Servant and Professional: Understanding the Challenges of Being A Public Service Doctor in A Plural Health Care Setting in Rural South Africa [thesis]. Pretoria: University of Pretoria; 2014.

[17] Aitken JM (1994). Voices from the inside: managing district health services in Nepal. Int J Health Plann Manage.

[18] Ramani S, Sivakami M, Gilson L (2018). How context affects implementation of the Primary Health Care approach: an analysis of what happened to primary health centres in India. BMJ Glob Health.

[19] Peabody JW, Taguiwalo MM, Robalino DA, Frenk J. Improving the quality of care in developing countries. In: Jamison DT, Breman JG, Measham AR, et al, eds. Disease Control Priorities in Developing Countries. 2nd ed. Washington, DC: The International Bank for Reconstruction and Development, The World Bank; 2006. 21250362

[20] Madhiwalla N, Ghoshal R, Mavani P, Roy N (2018). Identifying disrespect and abuse in organisational culture: a study of two hospitals in Mumbai, India. Reprod Health Matters.

[21] Kahabuka C, Moland KM, Kvåle G, Hinderaker SG (2012). Unfulfilled expectations to services offered at primary health care facilities: experiences of caretakers of underfive children in rural Tanzania. BMC Health Serv Res.

[22] Audo MO, Ferguson A, Njoroge PK (2005). Quality of health care and its effects in the utilisation of maternal and child health services in Kenya. East Afr Med J.

[23] Government of India. Bulletin of Rural Health Statistics. New Delhi: Ministry of Health and Family Welfare, Government of India; 2017.

[24] Government of India. Indian Public Health Standards. Revised guidelines. New Delhi: Ministry of Health and Family Welfare, Government of India, 2012.

[25] Government of India. The Planning Commission. High Level Expert Group on Universal Health Coverage. New Delhi: Government of India; 2011.

[26] Medical Council of India. https://www.mciindia.org/. Accessed February 16, 2019.

[27] National Health Systems Resource Centre (NHSRC). A Review of Existing Regulatory Mechanisms to Address the Shortage of Doctors in Rural, Remote and Underserved Areas. http://nhsrcindia.org/sites/default/files/Regulatory%20study%20report.pdf. Published 2016.

[28] Ranjan A. Measuring Equity as A Dimension of Progress Towards Universal Health Coverage [thesis]. Mumbai: Tata Institute of Social Sciences; 2017.

[29] Rao KD, Shahrawat R, Bhatnagar A (2016). Composition and distribution of the health workforce in India: estimates based on data from the National Sample Survey. WHO South East Asia J Public Health.

[30] Gilson L, Schneider H, Orgill M (2014). Practice and power: a review and interpretive synthesis focused on the exercise of discretionary power in policy implementation by front-line providers and managers. Health Policy Plan.

[31] Brehm JO, Scott G. Working, Shirking, and Sabotage: Bureaucratic Response to a Democratic Public. Ann Arbor: University of Michigan Press; 1999.

[32] Tummers LLG, Bekkers V, Vink E, Musheno M (2015). Coping during public service delivery: a conceptualization and systematic review of the literature. J Public Adm Res Theory.

[33] Gore RJ. Market Reform, Medical Care, And Public Service: Dilemmas of Municipal Primary Care Provision in Urban India [thesis]. New York City: Columbia University; 2017.

[34] Gofen A (2014). Mind the gap: dimensions and influence of street-level divergence. J Public Adm Res Theory.

[35] Stone CN (1983). Whither the welfare state? professionalization, bureaucracy, and the market alternative. Ethics.

[36] Erasmus E, Gilson L, Govender V, Nkosi M (2017). Organisational culture and trust as influences over the implementation of equity-oriented policy in two South African case study hospitals. Int J Equity Health.

[37] Belaid L, Ridde V (2015). Contextual factors as a key to understanding the heterogeneity of effects of a maternal health policy in Burkina Faso?. Health Policy Plan.

[38] Okello G, Molyneux S, Zakayo S, Gerrets R, Jones C (2019). Producing routine malaria data: an exploration of the micro-practices and processes shaping routine malaria data quality in frontline health facilities in Kenya. Malar J.

[39] Evans T (2011). Professionals, managers and discretion: critiquing street-level bureaucracy. Br J Soc Work.

[40] Cohen N (2018). How culture affects street-level bureaucrats’ bending the rules in the context of informal payments for health care: the Israeli case. Am Rev Public Adm.

[41] Hipgrave DB, Hort K (2014). Dual practice by doctors working in South and East Asia: a review of its origins, scope and impact, and the options for regulation. Health Policy Plan.

[42] Government of India. Report of the Task Force on Comprehensive Primary Health Care Rollout. New Delhi: Ministry of Health and Family Welfare, Government of India; 2014-2015.

[43] Sundararaman T, Gupta G (2011). Indian approaches to retaining skilled health workers in rural areas. Bull World Health Organ.

[44] Ramani S, Rao KD, Ryan M, Vujicic M, Berman P (2013). For more than love or money: attitudes of student and in-service health workers towards rural service in India. Hum Resour Health.

[45] Purohit B, Bandyopadhyay T (2014). Beyond job security and money: driving factors of motivation for government doctors in India. Hum Resour Health.

[46] Gilson L (2016). Everyday politics and the leadership of health policy implementation. Health Syst Reform.

[47] Lehmann U, Gilson L (2015). Action learning for health system governance: the reward and challenge of co-production. Health Policy Plan.

[48] Mbindyo P, Gilson L, Blaauw D, English M (2009). Contextual influences on health worker motivation in district hospitals in Kenya. Implement Sci.

[49] Munabi-Babigumira S, Glenton C, Willcox M, Nabudere H (2019). Ugandan health workers’ and mothers’ views and experiences of the quality of maternity care and the use of informal solutions: a qualitative study. PLoS One.

[50] Alonso-Garbayo A, Raven J, Theobald S, Ssengooba F, Nattimba M, Martineau T (2017). Decision space for health workforce management in decentralized settings: a case study in Uganda. Health Policy Plan.

[51] Gross K, Armstrong Schellenberg J, Kessy F, Pfeiffer C, Obrist B (2011). Antenatal care in practice: an exploratory study in antenatal care clinics in the Kilombero Valley, south-eastern Tanzania. BMC Pregnancy Childbirth.

[52] Zarychta A, Grillos T, Andersson KP (2020). Public sector governance reform and the motivation of street-level bureaucrats in developing countries. Public Adm Rev.

